# The role of hand size in body representation: a developmental investigation

**DOI:** 10.1038/s41598-022-23716-6

**Published:** 2022-11-11

**Authors:** Dorothy Cowie, Janna M. Gottwald, Laura-Ashleigh Bird, Andrew J. Bremner

**Affiliations:** 1 Department of Psychology, Durham University, Durham, UK; 2 Department of Psychology, Uppsala University, Uppsala, Sweden; 3 Centre for Developmental Science, School of Psychology, University of Birmingham, Birmingham, UK

**Keywords:** Human behaviour, Psychology, Sensory processing

## Abstract

Knowledge of one’s own body size is a crucial facet of body representation, both for acting on the environment and perhaps also for constraining body ownership. However, representations of body size may be somewhat plastic, particularly to allow for physical growth in childhood. Here we report a developmental investigation into the role of hand size in body representation (the sense of body ownership, perception of hand position, and perception of own-hand size). Using the rubber hand illusion paradigm, this study used different fake hand sizes (60%, 80%, 100%, 120% or 140% of typical size) in three age groups (6- to 7-year-olds, 12- to 13-year-olds, and adults; *N* = 229). We found no evidence that hand size constrains ownership or position: participants embodied hands which were both larger and smaller than their own, and indeed judged their own hands to have changed size following the illusion. Children and adolescents embodied the fake hands more than adults, with a greater tendency to feel their own hand had changed size. Adolescents were particularly sensitive to multisensory information. In sum, we found substantial plasticity in the representation of own-body size, with partial support for the hypothesis that children have looser representations than adults.

## Introduction

Perceiving one’s own body is fundamental to human experience, both for acting skillfully in our environments and for distinguishing between oneself and others. Yet own-body representation is complex^1^, involving the integration of prior knowledge about the body with incoming sensory inputs across multiple senses^2^. An important type of prior knowledge concerns the visual sizes of one’s body and limbs. This is particularly important to successfully move with respect to goals and obstacles in the environment. There are also indications that limb size affects adults’ sense of embodiment of a limb, suggesting that adults have prior expectations about it^3^. However, unlike other key visual cues to embodiment, body sizes—as well as sensory systems—change considerably over development^4^. This therefore raises questions about how such cues develop. The current study undertook a systematic investigation of how hand size affects hand embodiment and action-oriented judgments across childhood, early adolescence, and adulthood. We tested the hypothesis that hand size places less constraint on experiences of embodiment earlier in life, becoming an increasingly important constraint with age and experience. This would allow young children a greater level of acceptance of different hand sizes in the context of rapid physical growth, while providing adults with useful constraints on what they embody.

Childhood sees broad shifts in how sensory information is used to establish a sense of body ownership. While children from around 5–10 years of age use multisensory cues to identify and localize body parts^5–10^, they also show much stronger responses to bodily illusions than adults^6^. This likely results from high visual capture, such that correctly-oriented, body-like, objects in near space tend to be perceived as one’s own^5,9^ Young children are not only subject to these sensory changes, but to rapid and significant bodily growth (~ 1.3 cm in hand length per year^6^). This necessitates frequent recalibration between seen and felt hand positions^11^, until hand growth slows and stabilizes in the teenage years (the discrepancy between average 12–13-year-old hands and adults’ is less than 1cm^6^).

How, during such significant sensory and bodily changes, could a child use hand size to perceive and identify their own body? It might function as a high-level constraint such that prior expectation regarding hand size shapes the processing of incoming sensory information about nearby body parts^12^. Indeed, for competent motor control alone, one would expect children to have some expectations regarding own-hand size. On the other hand, one might predict a degree of plasticity in children’s hand representation. Since hands grow but do not shrink, one might expect participants to accept larger but not smaller hands than their own^13^. Particularly around 5–10 years of age, because of the sensory changes and bodily growth detailed above, there might be enhanced plasticity. This would allow children to accept their growing hand as their own; it would also make them more susceptible to illusions of ownership over differently-sized hands. In summary, one might expect some plasticity in hand size representation at all ages, and for children to represent hand size more loosely than adolescents or adults.

To examine hand size as a cue to embodiment, many studies have used versions of the classic Rubber Hand Illusion (RHI), in which adults and children from 4–5 years of age report that a fake hand feels as if it is their own when they view stroking on it as they feel strokes on their own, hidden, hand^5,14–16^. Across a few minutes, a “drift” in perceived hand location towards the fake hand grows, along with a more explicit sense that it is part of the participant’s body (“ownership”). Crucially, if the appearance of the fake hand is altered, the illusion is reduced^17,18^, suggesting that prior expectations about body form gate incoming sensory information^14^. While there have been some recent challenges to this paradigm which appeal to the demand characteristics of the illusion^19^, these do not seem able to account for all of the reported effects^20^.

RHI studies with adults reveal a need for more work on how hand size may constrain embodiment. Some work shows that adults can embody larger, but not smaller, hands—in terms of drift^3^, changes to grip aperture^21^, or the perception of tactile stimuli^22^. This pattern of findings is termed the ‘rubber band hypothesis’ given the analogy that both limb representations and rubber bands can more easily be stretched than compressed^22^. There is however scant data from these studies on how hand size affects hand *ownership*. Further, some reports showed that tactile stimulus judgments^23^ or ownership ratings^24^ can be altered by *either* large or small fake hands. Finally, irrespective of how hand size may initially constrain ownership over a fake hand, feeling ownership over a larger fake hand may change the perceived size of one’s *own* hand^24^. In sum, to understand how hand size constrains embodiment in adults there is a need for more data on explicit ownership and its relation to subjective reports of hand size. Further, to reveal any more nuanced tuning function between hand size and ownership, there is a need for studies to use a range of hand sizes rather than simply one ‘large’ or ‘small’ hand.

Some work has investigated how children perceive and embody hands of different sizes. When asked to visually match the size of their own hidden hand to a viewed hand^4,25^, children substantially underestimated their hand size compared to adults. For 8–15-year-olds presented with illusory finger growth through a *Mirage* box, over 80% agreed with the statement that their finger had “really stretched” (^26^, see also^27^). An RHI study with 6–8-year-olds found somewhat equivocal results: high ownership for both medium and large fake hands^9^, but marginally higher drift in perceived hand location for a medium hand. In the absence of additional hand sizes or ages, we cannot draw wider conclusions from this study regarding the more specific effects of hand size and age on embodiment (for example, whether any difference in drift was due to a *differently*-sized hand or specifically a *larger* hand; whether or not children are similar to adults). A large study^12^ found feelings of ownership over both small and large fake hands; however, different ages received proportionally different fake hand sizes and there was no comparison medium hand. Their result that ownership over a small hand decreased with age strongly motivates further targeted developmental work. Finally, this study demonstrates the strength of action-based measures of perceived hand size following the RHI: participants of all ages judged they could fit their hands through larger spaces following an RHI with a large fake hand.

Taken together, these developmental studies are consistent with the hypothesis that children more readily embody differently-sized hands than adults, but also clearly illustrate the need for additional systematic investigation. We suggest that further work should use a range of both smaller and larger fake hand sizes, scaling in proportion to hand size at each age. There is also a need for more systematic investigation of age effects. From the results reviewed above, we know that children of 6–9 years respond more strongly to the basic RHI than older children or adults^5^. Further, at this age the hand is still growing at over 1 cm per year. Children of this age are therefore an important group to test, and it seems sensible to focus on the youngest age band within this, 6–7 years, since this is the most likely to differ from adults. By 12–13 years, hand growth has slowed to < 1 cm per year and sensory integration is adultlike^6,11,28^. This group is therefore also important, as it may be on the cusp of adult-like responses. As noted, further work is also needed on adults. In sum, sampling at 6–7 years, 12–13 years, and adults should reveal any developmental progression from the period of rapid sensory and physical change in mid-childhood through to a more adultlike state in early adolescence; as well as the full adult profile.

Here we report such a RHI study in which we compared embodiment of a range of different sizes of fake hands in 6–7-year-olds, 12–13-year-olds and adults. At each age, we compared, between-subjects, the effect of five hand sizes, representing 60, 80, 100, 120 and 140% of mean hand size for that group, as measured from previous samples^6^. By testing this series of graded sizes, we hoped to reveal any tuning function between hand size and embodiment. By examining three age groups, we tested whether and how this tuning might change with age. To examine how prior expectations regarding hand size might interact with visuotactile multisensory information, we compared responses across synchronous and asynchronous stroking conditions. We measured ownership over a fake hand, touch referral to it, and the subjective sense of whether one’s own hand had changed size^24^. Alongside these questions^6,8,9^, we took action-oriented judgements following the illusion: the drift in participants’ perceived hand location towards the fake hand^5^, and the perceived size of the hand in an affordance judgment task^12^.

We interrogated the data according to several key questions. First, we examined the extent to which the different age groups showed tuning of our measures to hand size: specifically, whether the influence of the fake hand declined with greater size discrepancies between the fake and real hand. We hypothesized that the precision of this tuning would increase with age. Next, we tested the claim arising from the literature^22^ that embodiment would be stronger for fake hands which are own-sized or larger than for those which are smaller. Finally, we expected that visuotactile synchrony would effect our measures at all ages and that visual capture of hand position would be greatest at 6–7 years (as in^5,6^).

## Methods

In this experiment we examined the effects of three factors on the embodiment of a fake hand. Age, a between-subjects factor, had 3 levels (6–7-year-olds, 12–13-year-olds, and adults). Hand size, a between-subjects factor, had 5 levels (XS, S, M, L, XL: details below). Synchrony, a within-subjects factor, had two levels (synchronous and asynchronous: details below).

**Figure 1:**
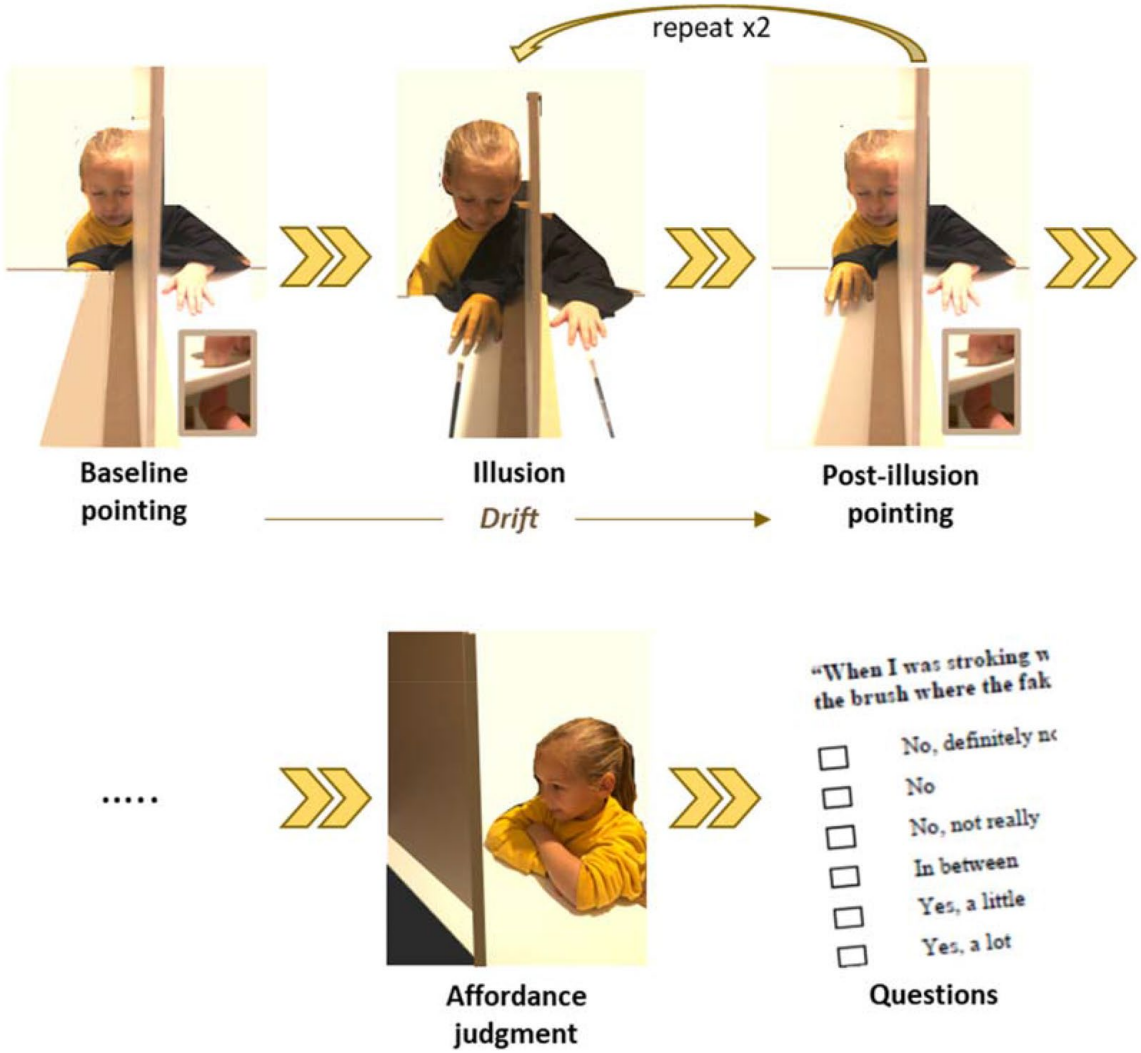
Procedure. *Baseline*: eyes closed, pointing under table to hand (inset). *Illusion*: stroking with brushes. *Post-illusion pointing*: eyes closed, pointing under table to hand (inset). Illusion & post-illusion points repeat (see “Methods”). *Affordance*: judging the size of an aperture the hand can fit through. Questions (see “Methods”). Fake hand for illustration only—see Fig. 2 for actual hands used.

All participants were recruited and tested in the U.K. Eighty-one 6- to 7-year-olds (45 girls, 36 boys, mean age 7.14 years, SD 0.31) and 77 12- to 13-year-olds (49 girls, 28 boys, mean age 13.16 years, SD 0.49) were recruited through local schools and our departmental database of local volunteer families. Seventy-five adults (48 women, 27 men, age range 18–54 years, mean age 22.14 years, SD 6.07) were recruited through the university’s study participant pool and word-of-mouth communication. The data of two 6–7-year-olds and two 12- to 13-year-olds were excluded due to inattentiveness, a reported ASD diagnosis, or monocular vision. All included data therefore came from participants with normal or corrected-to-normal vision, and with no known sensory, neurological or neurodevelopmental problems. Before testing, informed consent was obtained from the adult participants, and from the parents of child participants. For the child shown in Fig. 1, we obtained informed consent for the publication of identifying information/images in an online open-access publication. All experiments were approved by the Durham University Psychology Department Ethics committee (project reference 16/01) and were in accordance with the 1964 Helsinki declaration and its later amendments (World Medical Association, 2013).

We used a very similar procedure as in previous RHI studies with children^5,8^. At the beginning of each trial, the participant had their right hand placed under the table to their right side. The distance between their hand and their body’s midline was 50% of their arm length. (Throughout the procedure, we used each participant’s arm length to scale the setup, to keep motor demands constant for participants of different body sizes).

The experiment started with a training phase. Participants were asked to place their left hand on the table, roughly at body midline. Then they were asked to slide their right index finger horizontally along a groove under the table to point underneath their left index finger. This was done to train participants to point by sliding the finger, without looking, and to check that points were roughly underneath their finger.

We continued (Fig. 1) with two baseline trials. The left hand was placed on the table at a distance of 25% arm length from body midline. A screen was placed in between body midline and the left hand, so that it blocked the participant’s view of the left hand. The participant was asked to close their eyes, and again point with their right index finger underneath their left index finger. An experimenter marked the participant’s pointing position on a strip of paper underneath the table. This baseline procedure was repeated for a second trial. After this, the participant was asked to give the experimenter a “high-five” with their left hand: this was designed to reduce possible carry-over motor memory effects regarding hand position from baseline to the subsequent test trials.

**Figure 2:**
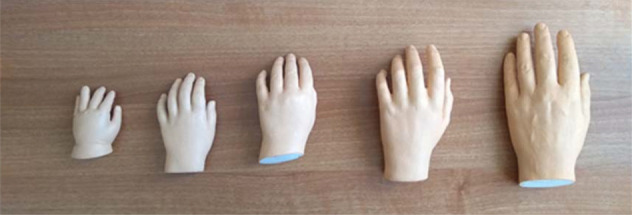
Fake hands. To illustrate the appearance of the fake hands, this figure shows the set used for 6–7-year-olds. A corresponding second set was used for the other two groups.

There were two further experimental blocks: one where visuotactile information was synchronous and one where it was asynchronous. The order of these was counterbalanced across participants. In each block, a plaster-cast fake hand, painted a light skin colour, was placed in front of the participant at their body midline. The size of the fake hand was varied across participants. The hand was either 60%, 80%, 100%, 120% or 140% of their age group’s average hand size, as measured from previous datasets^6^: these were labelled as XS, S, M, L, and XL respectively. Again based on previous datasets, the 12–13-year-old group were given the same set of fake hand sizes as the adults, while the 6–7-year-olds were given a smaller set of fake hands. The range of sizes is shown in Table 1 and illustrated for the 6–7-year-olds in Fig. 2. We used plaster-cast hands cast from real hands, except for the largest, which was 3D-printed.

**Table 1:** Fake hand sizes.

Fake hand size (%age of mean size for age group) and size ‘label’	Sizes for 6–7-year-olds (cm)	Sizes for 12–13-year-olds and Adults (cm)
60% (‘XS’)	7.5	10.5
80% (‘S’)	10.0	14.0
100% (‘M’)	12.5	17.5
120% (‘L’)	15.0	21.0
140% (‘XL’)	17.5	24.5

This table shows the set of fake hands given to each group of participants. The metric sizes here are labelled throughout the text as shown in the leftmost column. Size is the left hand length, measured with the hand flat and palm-up, from the wrist crease to the end of the middle finger.

Each block contained three test trials. On each of these, the participant closed their eyes and placed their hands at the same locations as at baseline. A cloth was placed to cover the left shoulder, left arm and the empty space between the fake hand and the body. The participant opened their eyes and observed the experimenter stroking the fake hand and the real hand with paintbrushes for 2 min. During the synchronous stroking condition, the experimenter stroked the real and fake hands at the same time and in the same location (e.g. upper index finger). During the asynchronous condition, the experimenter alternated strokes on the real and fake hands, stroking at different times and in different locations. In both conditions, strokes were given at roughly 1 Hz, and were short ‘dabs’ which covered a very small area of the finger. This was done to equalize the amount of sensory information available in each condition, since stroking the whole or half length of the finger would have resulted in much longer strokes for larger hands. After stroking had finished, the participant was asked again to close their eyes and point underneath their left index finger. This was repeated for two shorter trials, each containing a 20-s period of stroking followed by a point.

With the participant’s left hand remaining on the table, and their right hand in their lap, the affordance task was performed. We placed a simple device on the table in front of them, in which a screen could be drawn upwards to reveal a gap. We asked them to imagine giving a high-five with their upright left hand. As we slowly (~ 1 cm/s) increased the size of the gap, the participant was asked to say ‘stop’ when their left hand would *just* fit through the gap. We noted the size of the gap without providing feedback to them.

Thereafter, participants answered the following four questions: 1. “When I was stroking with the paintbrush, did it sometimes seem as if you could feel the touch of the brush where the fake hand was?”, 2. “When I was stroking with the paintbrush, did you sometimes feel like the fake hand was your hand, or belonged to you?”, 3. “When I was stroking with the paintbrush, did you sometimes feel like your own hand got bigger?” and 4. “When I was stroking with the paintbrush, did you sometimes feel like your own hand got smaller?”. The answer scale (coded from 0 to 6) was: “No, definitely not”/ “No”/ “No, not really”/ “In between”/ “Yes, a little”/ “Yes, a lot”/ “Yes, lots and lots”.

At the end of the first experimental block, participants were asked to pick a sticker from a box, with the hand movement preventing motor memory from one block to the next. The second block (synchronous/ asynchronous) was then performed.

### Ethical approval

All procedures performed were in accordance with the ethical standards of the regional ethics committee (assessment number Psychology 16/01) and with the 1964 Helsinki declaration and its later amendments or comparable ethical standards.

### Informed consent

Informed consent was obtained from the parents of all individual child participants included in the study, and from all individual adult participants.

## Results

### Analysis overview

We first present the responses to the questionnaire items, then the action-oriented measures of the aperture task and proprioceptive drift. Before submitting our ordinal questionnaire data to ANOVA, we used an Aligned Rank Transform procedure^29^. This method provides a bridge between parametric and non-parametric testing, by aligning and ranking raw data which can then be submitted to standard ANOVA. Importantly, this allows us to test for the presence of interactions in the data. For each questionnaire item, we conducted two ANOVAs on the aligned, ranked data. The first, ‘omnibus’, ANOVA examined the factors Hand Size (XS, S, M, L, XL), Synchrony (Synchronous, Asynchronous), and Age (Child, Adolescent, Adult). The second, ‘directed’ ANOVA tested the hypothesis that actual-sized or larger hands would more easily embodied than smaller, by grouping hand size into two levels. The factors for this are therefore Hand Size (XS, S vs. M, L, XL), Synchrony, and Age. From this directed analysis we do not repeat all effects but rather simply present the effects of interest, which are Hand Size and its interactions. For analyses of aperture judgments and proprioceptive drift we examine the effects of Hand Size, Synchrony and Age using standard ANOVA. For measures where we expected the participant’s response to change linearly as a function of fake hand size (‘my hand felt bigger’, ‘my hand felt smaller’, affordance task), we report the linear contrasts of Hand Size as well as its main effect.

### Question: touch referral to fake hand

**Figure 3:**
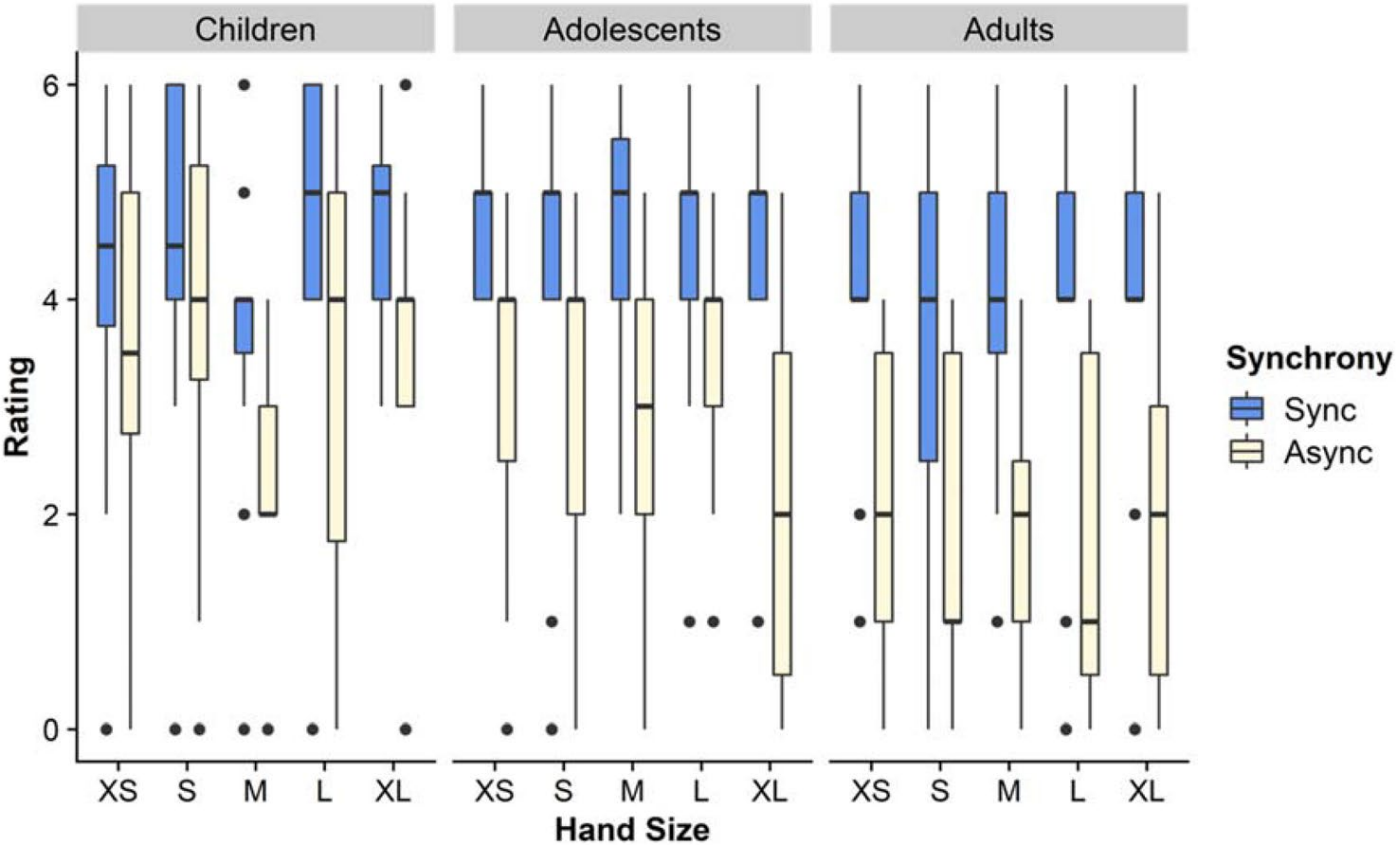
Touch referral. This figure shows the medians and interquartile ranges of ratings on the questionnaire item regarding touch referral to the fake hand. Ratings are shown for each age group, fake hand size, and condition.

In the omnibus analysis for this item (Fig. 3), we found a main effect of Age, (F(2,214) = 14.2, *p* < .001, η_p_^2^ = 0.117), with post-hoc Tukey HSD tests showing that both children (M = 3.72; *p* < .001) and adolescents (M = 3.69; *p* > .001) gave higher ratings than adults (M = 2.89). In addition, we found an effect of Synchrony, (F(1,214) = 201.5, *p* < .001, η_p_^2^ = 0.485), such that mean scores were higher for the synchronous (M = 4.22) than the asynchronous (M = 2.65) condition. Finally, we found an Age by Synchrony interaction, (F(1,214) = 201.5, *p* < .001 η_p_^2^ = 0.097. To follow this up, we used Sidak-corrected pairwise comparisons in a simple effects analysis. Since values from the Aligned Ranked Transform should not be used here^29^, this and subsequent simple effects analyses are based on raw questionnaire data. This analysis showed age-related changes in the asynchronous condition, such that responses were lower in this condition for adults than children, *p* = .001, and adolescents, *p* = .001. No other effects or interactions were significant. In the directed analysis, with smaller vs. larger hands, we found no effect of hand size or interactions of hand size with other factors (F’s < 2.317, *p*’s > .129).

### Question: ownership of fake hand

**Figure 4:**
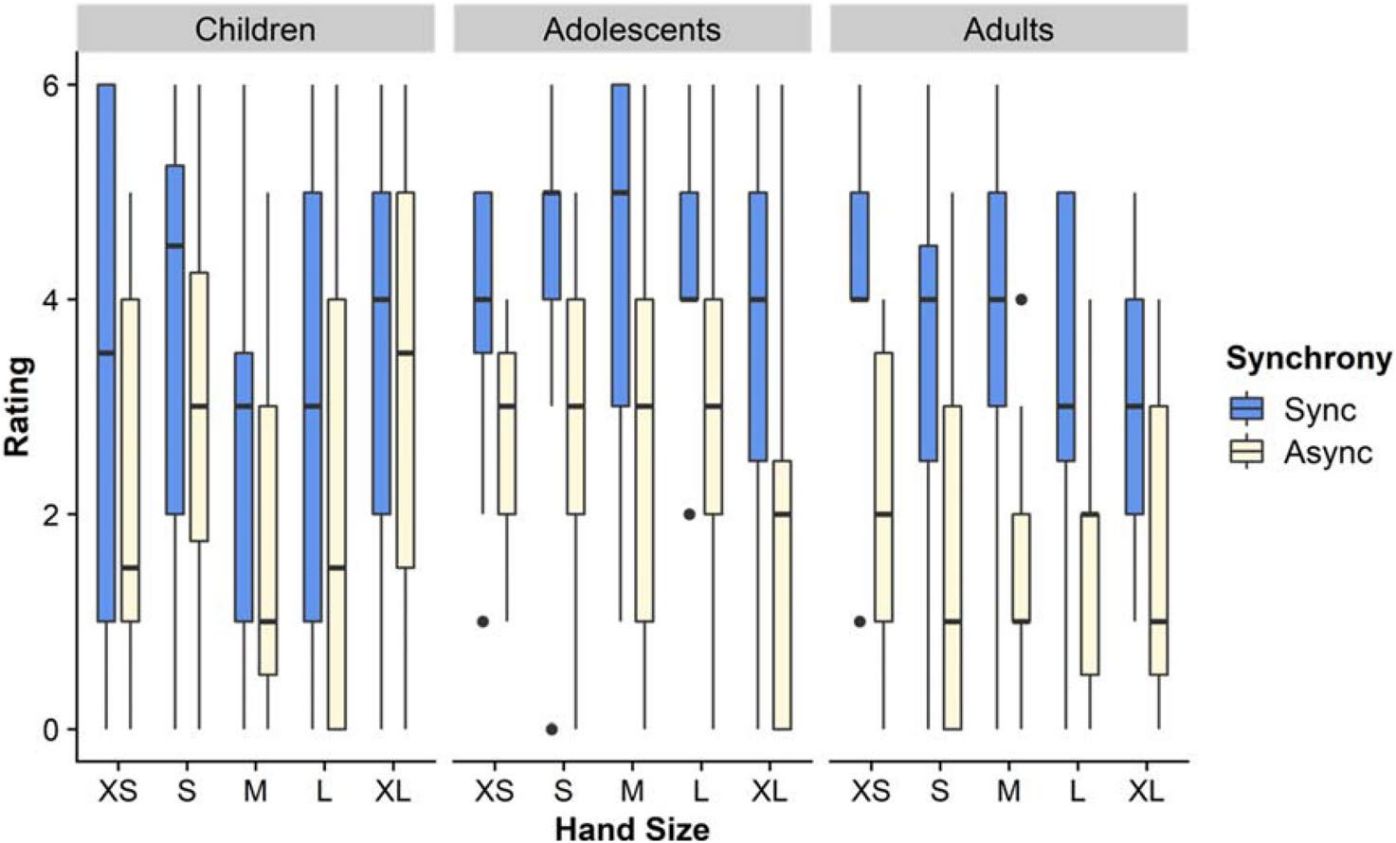
Ownership. This figure shows the medians and interquartile ranges of ratings on the questionnaire item regarding ownership of the fake hand. Ratings are shown for each age group, fake hand size, and condition.

In the omnibus analysis for this item (Fig. 4) we again we found a main effect of Age (F(2,214) = 4.185, *p* = .016, η_p_^2^ = 0.824). Adolescents (M = 3.29) gave higher ratings than adults (M = 2.64; *p* = .012), while children (M = 2.87) were not significantly different to either. We found an effect of Synchrony (F(1,214) = 201.5, *p* < .001, η_p_^2^ = 0.332), such that mean scores were higher for the synchronous (M = 3.63) than the asynchronous (M = 2.24) condition. Finally, we found an Age x Synchrony interaction (F(1,214) = 201.5, *p* < .001, η_p_^2^ = 0.054). To examine this, Sidak-corrected pairwise comparisons in a simple effects analysis showed that in the Synchronous condition children gave higher responses than adolescents, *p* = .032, while in the Asynchronous condition, adults gave lower ratings than either children, *p* = .008, or adolescents, *p* = .003. As for touch, therefore, the effects of age were more prominent in the asynchronous condition. No other effects or interactions were significant. In the directed analysis, with smaller vs. larger hands, we found no effect of hand size or interactions of hand size with other factors (F’s < 1.22 s, *p*’s > .270).

### Question: hand felt larger than usual

**Figure 5:**
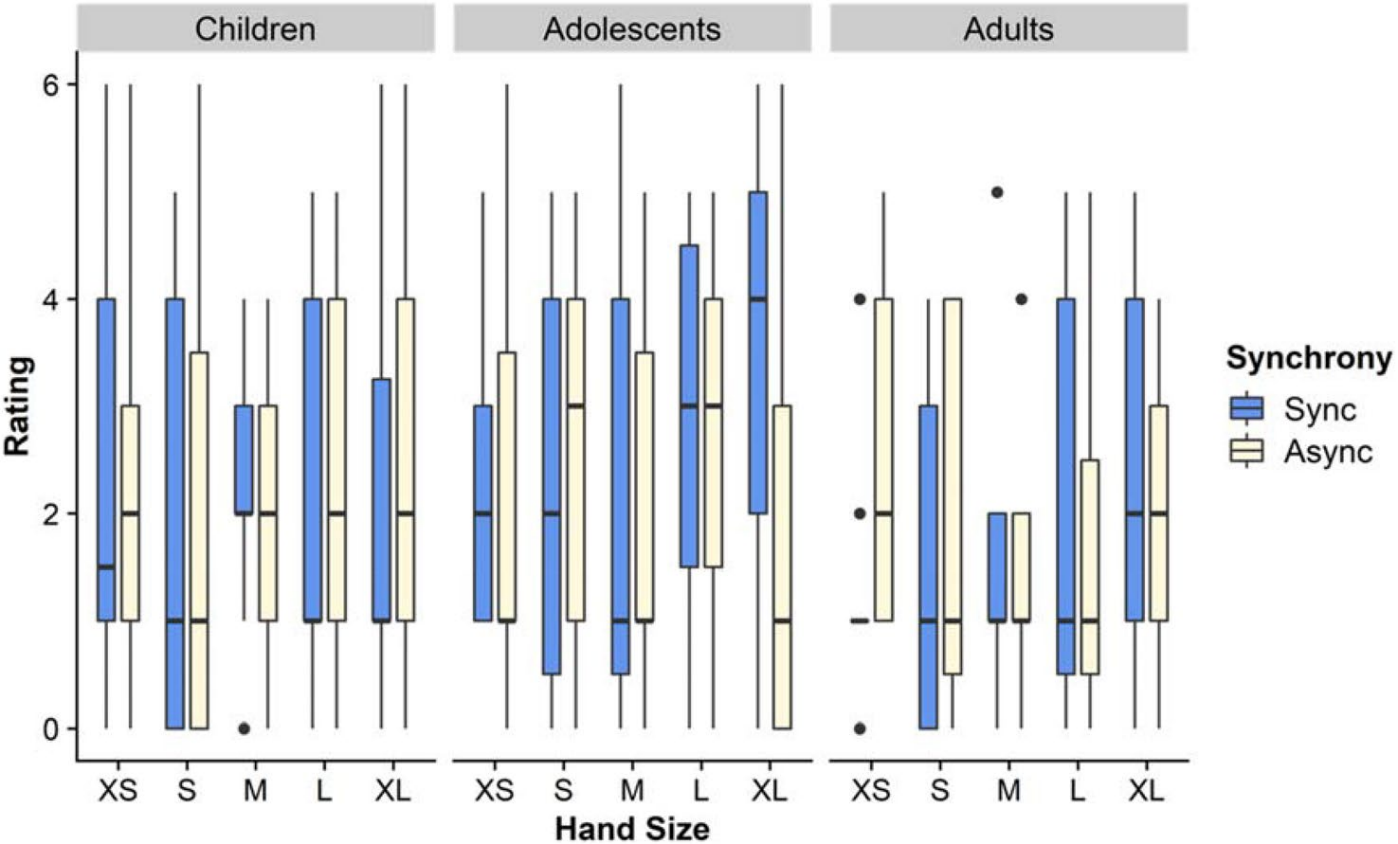
Hand felt larger. This figure shows the medians and interquartile ranges of ratings on the questionnaire item regarding one’s own hand feeling larger following the illusion. Ratings are shown for each age group, fake hand size, and condition.

In the omnibus analysis for this item (Fig. 5) we found an effect of Age (F(2,214) = 3.23, *p* = .042, η_p_^2^ = 0.029), such that adolescents (M = 2.46) gave higher ratings than adults (M = 1.81; *p* = .036), while children (M = 2.16) were not significantly different to either. No other effects or contrasts were significant. In the directed analysis with small vs. larger hands, we found no effect of hand size or interactions of hand size with other factors (F’s < 3.02, *p*’s > .084).

### Question: hand felt smaller than usual

**Figure 6:**
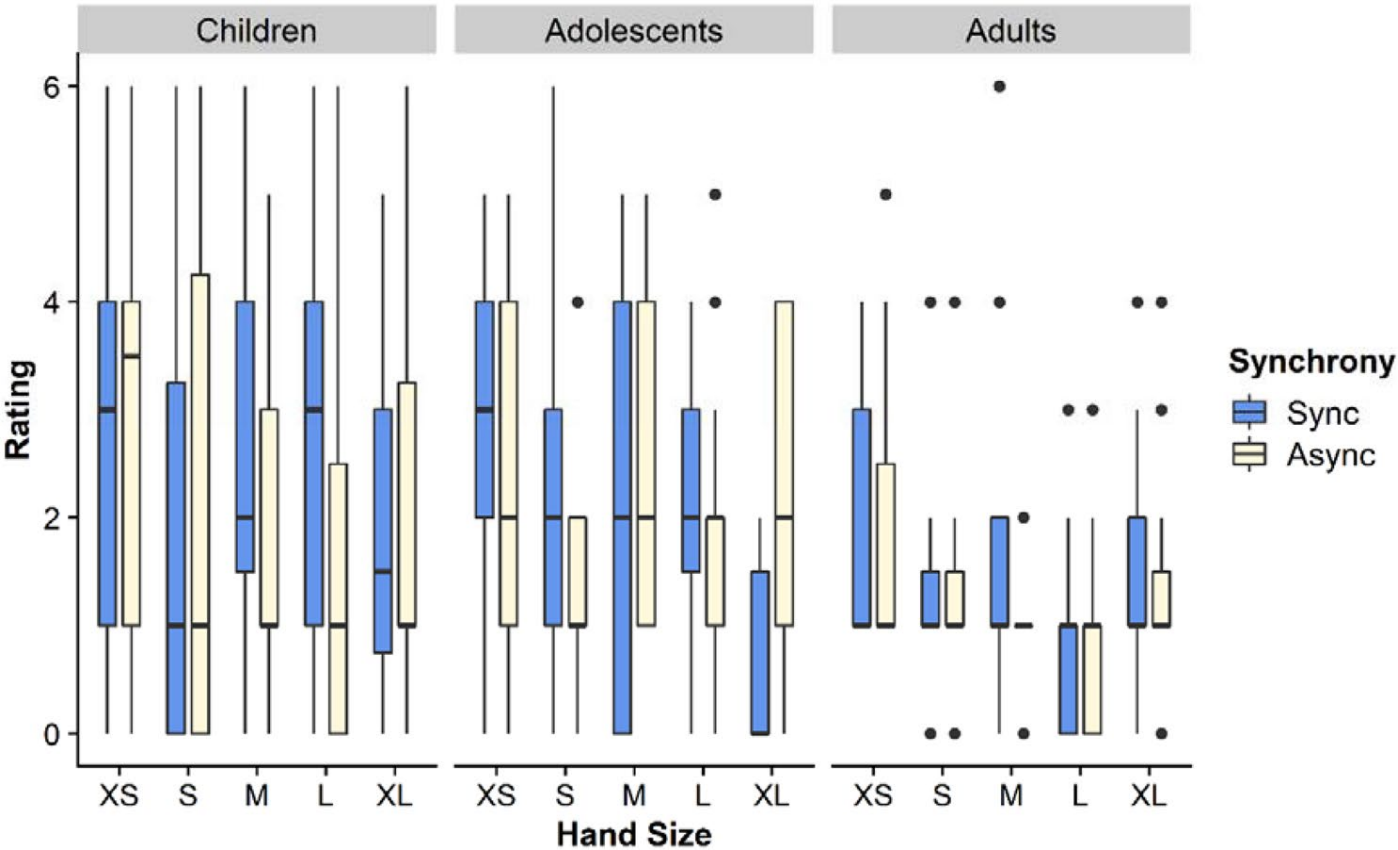
Hand felt smaller. This figure shows the medians and interquartile ranges of ratings on the questionnaire item regarding one’s own hand feeling smaller following the illusion. Ratings are shown for each age group, fake hand size, and condition.

In the omnibus analysis for this item (Fig. 6) we found an effect of Age (F(2,214) = 8.168, *p* < .001, η_p_^2^ = 0.071), such that children (M = 2.29; *p* = .001) and adolescents (M = 2.08; *p* = .002) gave higher ratings than adults (M = 1.39). In addition we found an effect of Hand Size (F(4,214) = 3.105, *p* = .016, η_p_^2^ = 0.055), with a linear contrast of *p* = .011, such that responses were higher in the XS condition (M = 2.47) than in the S (M = 1.74; *p* = .036) or XL (M = 1.64; *p* = .017) conditions.

Finally, we found a three-way interaction between Age, Hand Size and Synchrony, (F(8,214) = 2.229, *p* = .027, η_p_^2^ = 0.077), see Fig. 6. Examining this, Sidak-corrected pairwise comparisons in a simple effects analysis showed that for adolescents in the synchronous condition, responses were higher in the XS than in the XL condition, *p* = .002. Likewise for adolescents, responses were lower for synchronous stroking than asynchronous with the XL hand, *p* = .005.

These comparisons also showed that adults tended towards lower responses (less feeling that their hand was smaller) than other groups at larger hand sizes. Thus, adults’ responses were lower than adolescents’ in the medium hand-asynchronous condition *p* = .039 and large hand-synchronous condition, *p* = .035; and lower than children’s in the large hand-synchronous condition, *p* < .001. Finally, and contrary to prediction, we found that children showed higher responses (more feeling that their hand was smaller) in the synchronous than the asynchronous condition for the large hand, *p* = .018.

The directed analysis with small vs. larger hands, again revealed a three-way interaction between Age, Hand Size and Synchrony, (F(2,223) = 6.454, *p* = .002, η_p_^2^ = 0.055), but no other effects or interactions of Hand Size.

### Affordance judgments

**Figure 7:**
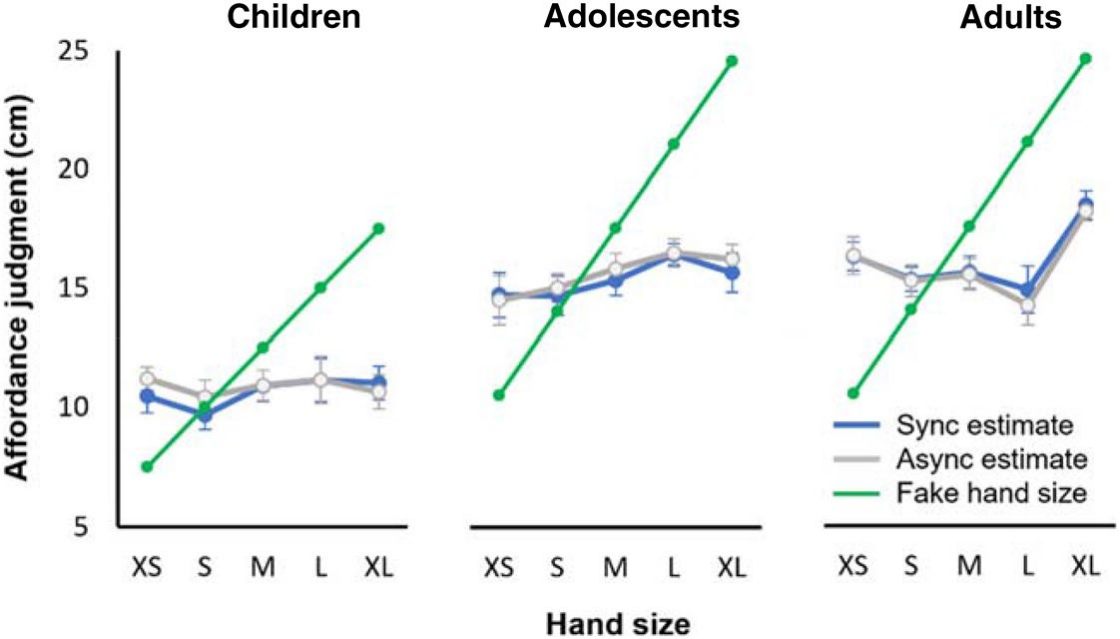
Affordance judgment. This figure shows the means and standard errors of affordance judgments, for each age group, fake hand size, and condition.

In this task the participant watched the experimenter slowly open a simple rectangular aperture between a base (small platform around eye level) and a thin sheet of a wood which lifted up (Fig. 1). The participant told them to stop opening it when they felt that their hand could just pass through. The resulting gap size is therefore an index of the participant’s estimated hand size following the illusion. The magnitude of the gap size (Fig. 7) was affected by Age, (F(2,214) = 89.849, *p* < .001), as would be expected given that children’s hands could fit through smaller gaps than adults’. While there was no main effect of Size, *p* = .064, the more specific linear contrast was significant, *p* = .021, such that the action-oriented estimate of one’s own hand size was smaller after viewing small fake hands and larger after viewing large fake hands.

Participants tended to underestimate hand size (scores were below zero). To quantify this, we calculated the discrepancy between each participant’s affordance estimate and their actual hand size, scaled as a percentage of each participant’s own-hand-size. On this measure we found an effect of Age only (F(2,214) = 7.348, *p* <  .001, η_p_^2^ = 0.064). Post-hoc Tukey HSD tests showed that, irrespective of stroking condition or hand size, children underestimated their hand size more than either adolescents (*p* = .002) or adults (*p* = .006).

### Proprioceptive drift

**Figure 8:**
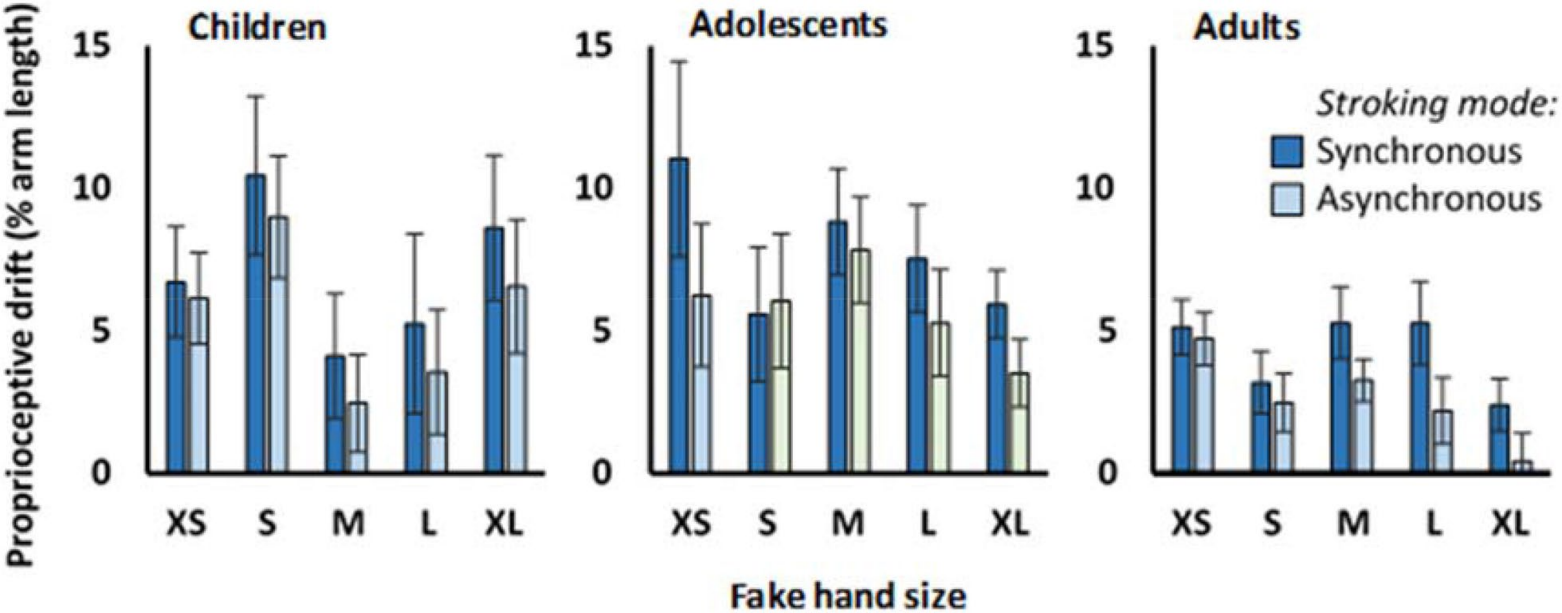
Proprioceptive drift. This figure shows the mean and standard errors of the proprioceptive drift measure, for each age group, fake hand size, and condition.

For this measure in the omnibus analysis (Fig. 8), we found an effect of Age, (F(2,214) = 4.938, *p* = .008, η_p_^2^ = 0.044). Post-hoc Tukey HSD comparisons showed higher drift in 6–7yo compared with adults (*p* = .032) and in 12–13yo compared with adults (*p* = .011). In addition, we found an effect of Synchrony, (F(1,214) = 16.58, *p* < .001, η_p_^2^ = 0.072), such that mean drift was higher for the synchronous than the asynchronous condition. The directed analysis for this measure likewise showed effects of Synchrony, (F(1,223) = 15.377, *p* < .001, η_p_^2^ = 0.072), and Age, (F(2,223) = 4.448, *p* = .013, η_p_^2^ = 0.038).

## Discussion

In this study we set out to examine whether children, young adolescents, and adults would embody hands of different sizes in the rubber hand illusion. All groups showed signatures of embodiment for all hand sizes, ranging from those which were 60% of their own hand size to those which were 140%. First, there were high ownership ratings and drift for the wide range of different fake hand sizes presented in the experiment, as well as effects of visuotactile synchrony on these measures, indicating that participants identified and localized these hands as their own. Second, both affordance judgments and ratings of own-hand size were modulated in a meaningful way by the size of the fake hand, indicating that embodiment of the fake hand changed the participant’s perception of their own hand. Together these indicate that hand size does not act as a high-level constraint on body representation but rather that there is significant plasticity in own-hand-size representation across ages.

Looking more specifically at ownership and drift, we found crucially that these were modulated very little by the size of the presented fake hands, with no evidence for any fine-grained tuning of ownership based on hand size. Rather, participants embodied hands of all sizes. More specifically, comparing ownership over hands that were smaller than one’s own with hands that were own-size or larger revealed little evidence to support the hypothesis that hand growth is easier to accept than shrinkage. This may be more specific to the scaling of tactile distance on the body^22,23^, rather than a general principle governing all aspects of embodiment including body ownership or touch referral.

Alongside changes to ownership following the illusion, we found changes to perceived hand size. In the action-oriented affordance measure participants judged their own hands as smaller after viewing a small fake hand, and larger after viewing a large fake hand, in agreement with^12^. Likewise on the questionnaire our participants reported that that their own hand felt smaller after viewing a small fake hand. This demonstrates substantial plasticity in own-body representation, such that incoming visual information can modulate long-term body representations. This complements findings from^24^, which used a visual matching task. Future work should determine how such different tasks may relate to one another as we seek to understand the interplay between hand ownership and perceived hand size.

Perceived hand size also changed with age. Children underestimated their own hand size significantly on the affordance task. This is consistent with^4^, who showed the same in a task where participants were asked to choose a viewed 3D hand model that matched theirs, and^25^, where underestimation was also found at 4–6 years using 2D images. However, the literature disagrees on the shape of developmental change. While Cardinali et al. show increasing underestimation between ages 6 and 10 years, Giurgola et al*.* show a similar underestimation in children and adults. The present study shows a third pattern, namely high underestimation in children and more veridical perception in adults. This issue should clearly be investigated in future studies.

Likewise, the propensity to feel that one’s hand had either shrunk or enlarged following the illusion was higher in younger age groups. Drawing together the affordance and questionnaire results on perceived hand size and age, it is possible that children’s inaccuracy and/or uncertainty regarding own-hand size, as revealed by the main effect of age on the affordance task, leaves them more susceptible to feeling their hand to be smaller following the illusion. In this way children’s inaccurate or unstable body representations may facilitate plasticity in own-body representation, such that hands of different sizes can be embodied.

Finally we note that the feeling of the hand being smaller in the extra-small condition was most pronounced for adolescents in the synchronous condition. Participants at this age may be particularly sensitive to the interplay between pre-existing constraints and incoming multisensory information, with visuotactile synchrony being used to strengthen the way in which pre-existing constraints shape the perception of hand size. While there has been relatively little work on bodily illusions in adolescence, the present results suggest that it might be an interesting age at which to examine body representation further.

Alongside these findings on hand size, we note several other important findings in the data. First, as predicted, drift in perceived hand location was larger at younger ages, we presume because the sight of the hand (irrespective of stroking) elicits a higher visual capture in young children^5,9^. In the present study, drift was also higher in adolescents than in adults. Some salient procedural differences between this study and the previous work on age-related changes in drift^6^ include more realistically-coloured hands, and smaller brushstrokes (see below). It is for future studies to determine the effect these may have on overall drift levels.

The second result we found unrelated to hand size was that, as predicted, there were consistent effects of visuotactile synchrony across ownership, touch referral and drift measures. The present study complements existing literature on the importance of touch for children as well as adults^5,6,8–10,30,31^, and adds to the growing movement to incorporate haptics as an essential element of virtually-controllable avatars. However, these effects of visuotactile synchrony were not consistent with age: in fact for touch referral and ownership, the role of synchrony grew with age. Specifically, responses decreased in the asynchronous condition. This contrasts with previous studies of the rubber hand illusion which revealed adult-like differences between synchronous and asynchronous conditions from 4 years^6,8^. Because the present study used identical questions and prompts to these previous studies, and because children of this age give very low ratings to control questions^7,32^, we argue strongly against explaining this result simply as a tendency for children of this age to avoid saying ‘no’.

Rather, we note that the present finding of a growing sensitivity to multisensory (a)synchrony with age is very similar to a study of the visuotactile full-body illusion in children^33^. Like this study, that one used strokes of short duration and short distance (‘dabs’), rather than the longer strokes in^5,6^. We suggest that the ‘dabs’ contain less multisensory information than the strokes, and that younger children are therefore less confident in using them to establish a sense of bodily self—and in particular, less confident in using them to *reject* a possible self in the asynchronous condition (by default, we may tend to embody a viewed body part^34^). While the visuotactile temporal binding window is larger at this age^35^, the gaps of ~ 1 s between our viewed and felt brush strokes and additional spatial asynchrony would have made these sensory discriminations relatively easy. However, this information may not yet be fed forward into the systems which establish a feeling of ownership over the body part. Examining directly the effects of different levels of visuotactile asynchrony would help illuminate this issue. In the interim, we conclude that children may use multisensory information differently to adults in establishing ownership over a body part, that the precise nature of this multisensory information may be important, and that these differences may persist into early adolescence.

The study has limitations which must be borne in mind when interpreting the results. In terms of the physical setup, it is possible that some limits to hand size would be found with a larger ranges of fake hand sizes—in extending a virtual arm, limits were found at 3–4 times its natural length^36^. Since they were cast from real hands, the fake sizes we used somewhat confounded hand age with hand size (the smallest hands in particular had notably chubbier fingers). We used only static hands—investigating moving bodies would provide a fuller picture of the representation of own-body size. In terms of measures, interpreting the main effects of age we found would have benefitted from the inclusion of control questions^19^, although we were able to draw on previous work to offset this issue. Likewise, we did not include the tactile distance perception measures that have been used in other studies^21,22^, nor ask participants to visually estimate their hand size^4,24,25^. It would have been useful to see how these related to the measures we took. A fuller picture would have been provided by testing whether illusory hand size scaled the perception of the surrounding visual environment^37,38^. Finally, we cannot assume that these results would necessarily translate to other body areas such as feet^39,40^; to whole-body illusions^41^; or to other sensory versions of the illusion^10^.

In conclusion, we found that participants were able to embody hands of a range of sizes, both smaller and larger than their own. This illusion also influenced the way in which hand size was experienced, with a small fake hand resulting in one’s own hand feeling smaller than usual and a large fake hand resulting in one’s own hand feeling larger. Both results suggest significant plasticity in the representation of one’s own hand size, across ages. Further, this plasticity was higher in children and adolescents, who had a greater tendency to rate their hand as having changed size. Early adolescents were more sensitive to the interplay of synchrony and size than other groups. We also found that participants all underestimated their own hand size, but that these distortions reduced with age. As well as these findings on size, we found that in this paradigm the effects of visuotactile synchrony grew with age. Children therefore use multisensory information differently to adults in establishing ownership over a body part, supporting recent theories^42^ that own-body representation develops over a prolonged developmental period, as a result of significant multisensory experience.

## Data Availability

Data are available on OSF at https://doi.org/10.17605/OSF.IO/X8K4G.
